# Epidemic history investigation: a new method of finding close contacts

**DOI:** 10.3389/fpubh.2023.1062633

**Published:** 2023-06-22

**Authors:** Xin Li, Yalan Li, Tianjiao Liu, Rui Ding, Qiannan Hou, Liling Xiong, Na Du, Zhaolin Gong, Linbo Cheng, Dan Luo, Sumei Wei, Xiao Yang

**Affiliations:** ^1^Chengdu Women’s and Children’s Central Hospital, School of Medicine, University of Electronic Science and Technology of China, Chengdu, China; ^2^The Fourth People’s Hospital of Chengdu, School of Medicine, University of Electronic Science and Technology of China, Chengdu, China; ^3^First Clinical College, Chongqing Medical University, Chongqing, China

**Keywords:** COVID-19, close contact, epidemic outbreak, epidemiology investigation, spacetime companion

## Abstract

**Introduction:**

Coronavirus disease 2019 has become a major global public health concern in December 2019. However, finding and excluding close contacts of COVID-19 infectors is a critical but difficult issue. This study aimed to introduce a new method of epidemiological investigation named space–time companions, which was adopted in Chengdu, China, in November 2021.

**Methods:**

An observational investigation was conducted during a small outbreak of COVID-19 in Chengdu, China in November 2021. A new method of epidemiological investigation called space–time companion was adopted in this outbreak, which was defined as the one who stayed in the same spatiotemporal grid (range: 800 m * 800 m) with the confirmed COVID-19 infector for more than 10 min in the last 14 days. A flow chart was used to describe the screening process of space–time companions in detail and illustrate the space–time companion epidemic management method.

**Results:**

The COVID-19 epidemic outbreak in Chengdu was effectively controlled for approximately one incubation period (14 days). After four rounds of space–time companions screening, more than 450,000 space–time companions were screened, including 27 COVID-19 infectors. Moreover, in the subsequent rounds of nucleic acid testing for all people in the city, no infected person were found proving the end of this epidemic outbreak.

**Conclusion:**

The space–time companion provides a new idea for screening close contacts of the COVID-19 infector and other similar infectious diseases, which can serve as a supplement to traditional epidemiological history surveys to verify and avoid missing close contacts.

## Graphical Abstract

Diagnosis diagram and processing flowchart of space–time companion. ^#^This concept map is adapted from social media networks.

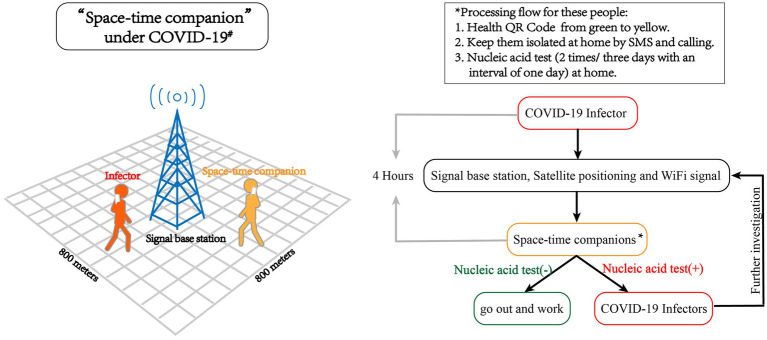

## Introduction

The rapid spread of the coronavirus disease 2019 (COVID-19) pandemic has become a major global public health concern since the first case of COVID-19 was diagnosed in December 2019 (1–3). Through active prevention and control measures, the prevalence of COVID-19 in China was effectively suppressed. However, during November 2021 to April 2022, with the start of winter and the delta variant, the prevalence of COVID-19 has shown a distinct upward trend (4, 5). This mutant strain has a 60% higher risk of community transmission than the alpha strain and could cause asymptomatic infection in vaccinated patients (6–9). Considering that this epidemic is sporadic and small-scale, which is different from the previous COVID-19 outbreaks, it is not suitable to take severe measures, such as nationwide nucleic acid detection or citywide blockade.

In addition, close contact between confirmed cases and personnel with epidemiological history and COVID-19 manifestations (fever, cough, and dizziness) should be identified efficiently and effectively in public places, especially in hospitals (10–12). However, the traditional methods of epidemiological history inquiry are laborious, time-consuming, and unable to detect misleading information (13–15). Most of the confirmed cases in this epidemic were infected with the delta variant. It has the potential to cause asymptomatic breakthrough infections in vaccinated patients, making it unfeasible to triage patients based on body temperature and COVID-19-like clinical manifestations.

Finding and preventing close contact with COVID-19 patients has been a critical issue since the outbreak of COVID-19 (16, 17). In the past, after confirming individuals infected with COVID-19, all people were isolated and the nucleic acid in the area was determined by the queried activity area and traffic route of the infectors in China (18–20). However, this method was mainly based on artificial epidemiological investigations and consumed a considerable amount of time and social resources (21, 22). Moreover, the results may be inaccurate due to omission and concealment, which can have serious consequences (23, 24). As a result, how to find and exclude close contacts in a more timesaving, labor-saving, and comprehensive way must be considered to prepare for a possible epidemic of mutated or other virus in the future.

Therefore, in the present study, we aimed to introduce a new method of epidemiological investigation called space–time companion, which was adopted in Chengdu, China in November 2021. This new communication network-based epidemiological history investigation method can actively discover potential close contacts and achieve a transition from subjective admission to objective discovery for close contacts. Given the findings reported in our study, we aimed to provide a new method for screening close contacts of individuals with COVID-19 and other similar infectious diseases.

## Materials and methods

### Space–time companion

The term space–time companion has received a special meaning since the small outbreak of the COVID-19 epidemic in Chengdu, China, on November 3, 2021. On November 3, 2021, a fever patient with a positive nucleic acid test result was found in Chengdu, China. The Chinese government immediately asked China Mobile, China Telecom, China Linkage, and other communication companies to provide communication data such as base stations, satellites, and Wi-Fi networks. Through a specific algorithm, “The Space–time companion is the one who stayed in the same spatiotemporal grid (range: 800 m * 800 m) with the confirmed COVID-19 infector for more than 10 min in the last 14 days.” The selected people were called space–time companions.

### Space–time companion management process

**Figure 1:**
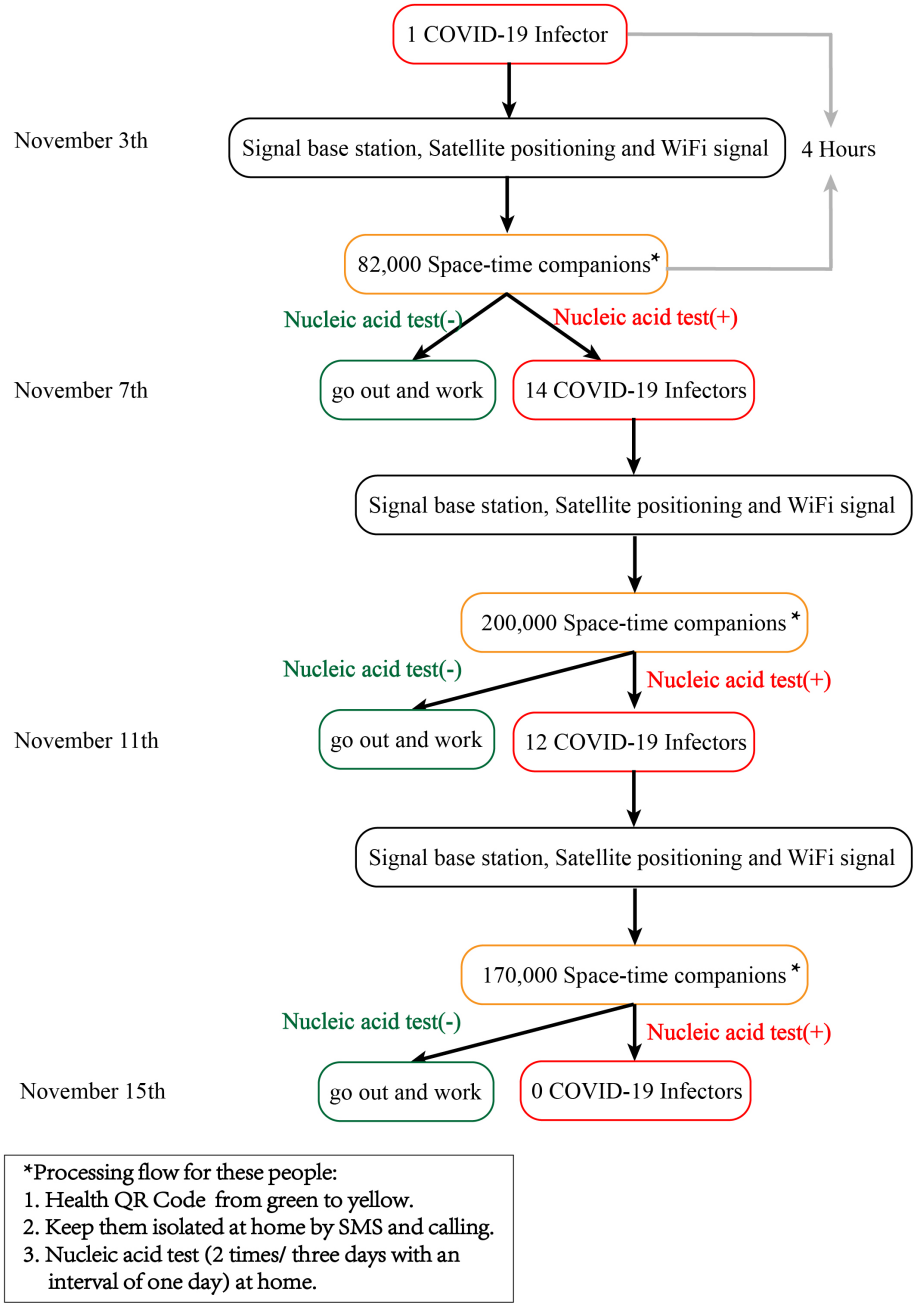
Process flow chart of people identified as space–time companions.

For space–time companions, the green Health QR Code quickly changed to yellow through the Health QR Code system (9, 25). The Health QR Code system and three-color circle-layer management system in Chengdu, China have been described in detail elsewhere (9). Telephone numbers and other personal information of the people were queried using the Chinese public security system. A short message about people who were identified as space–time companions and subsequent processing schemes was sent to their mobile phones *via* SMS. They were required to immediately return to the nearest place of residence and confirm the receipt of SMS. Those who did not confirm the SMS content were notified by telephone. If the phone could not be connected, the families were contacted. The medical staff called these people to isolate themselves at home, inquired about the epidemic history, asked for the current detailed address, and was informed about the treatment process of spatiotemporal companions. Space–time companions underwent nucleic acid testing at home by the medical staff and were asked if they had symptoms related to COVID-19. During the isolation period, community service personnel helped purchase living materials. After the nucleic acid test results (2 times/3 days with an interval of 1 day) were obtained, the health code of people with a negative nucleic acid test result changed back to green, and they continued to work. Individuals who tested positive were isolated by medical personnel and transported to the corresponding hospital for isolation and treatment. The next round of screening was conducted immediately after the detection of a newly infected person. The detailed process is illustrated in Figure 1. In addition, 3 days chosen as the time period for PCR testing is because previous studies have shown that the incubation period for Delta and Omicron variants is 3–4 days, while positive nucleic acid testing occurs slightly earlier than the incubation period (26, 27).

The space–time companion method for investigating epidemic history is based on the three-color Health QR Code system. This system is based on WeChat or Alipay applications, which were mandatory to be used during the epidemic. The three-color Health Code System require people use their mobile phones to show their green health code when entering any public place (such as a company or community), to prevent anyone from using shutdown methods to escape monitoring. This system can collect and save data such as everyone’s phone number, personal identification information, and movement trajectory. Of course, only government authorized epidemic prevention and control personnel can have restricted access to partial information.

### The health QR code system

The Health QR code is a tracing application bound with resident identity information to express that the user authorizes others or organizations to temporarily access specific personal health information. Relying on the mobile phone global positioning system (GPS) and the geographic location data of network operators to collect users’ space and time data, two-dimensional barcodes are used as storage media, which generally run on social media applications, such as WeChat and online payment platforms, such as Alipay. The system records and updates the users’ health information related to COVID-19, such as body temperature, clinical symptoms (fever, cough, dizziness, etc.), potential contact with infected patients, and daily travel details. To facilitate display and understanding, red, yellow, and green were used to represent health status. With the green health QR code, you can move freely in the city using public transportation; enter shopping malls, parks, and other places. A yellow or red health QR code indicates that the user has a medium or high exposure risk and will be required to undergo nucleic acid testing and quarantine.

### Data collection

We collected the number of new confirmed diagnoses and duration of COVID-19 from the official website of the National Health Commission of the People’s Republic of China,^1^ including the outbreak of November 3, 2021 in Chengdu epidemic.

## Results

**Figure 2:**
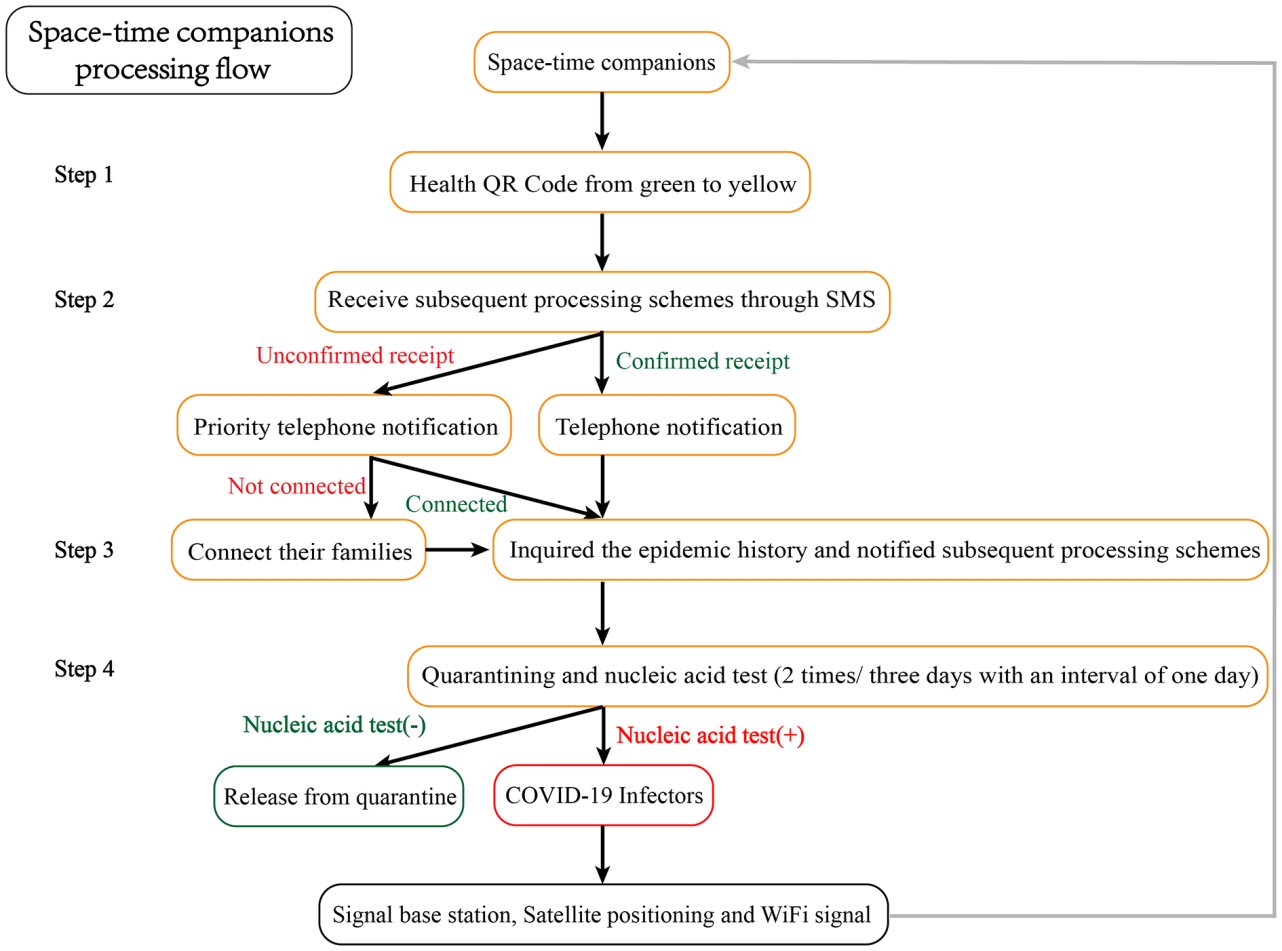
Flow chart and results of new epidemiological screening for the outbreak in Chengdu, China.

During this epidemic outbreak, 82,000 space–time companions were found within 4 h. As of November 7, 14 infectors had been found from the Chengdu population, and the screening scope was expanded according to the same method. In the second round of screening, nearly 200,000 people were identified as space–time companions, and they were isolated and observed at home in the same manner. As of November 11, 12 infectors were identified in the second round of screening, and the third round of screening was conducted accordingly. Until now, on November 15, no infected person was found in the third round of screening. As of November 24, no new infections had been found, and the Chengdu epidemic situation leading group announced the official end of the Chengdu epidemic situation the same day. The detailed process of the epidemic is shown in Figure 2.

**Figure 3:**
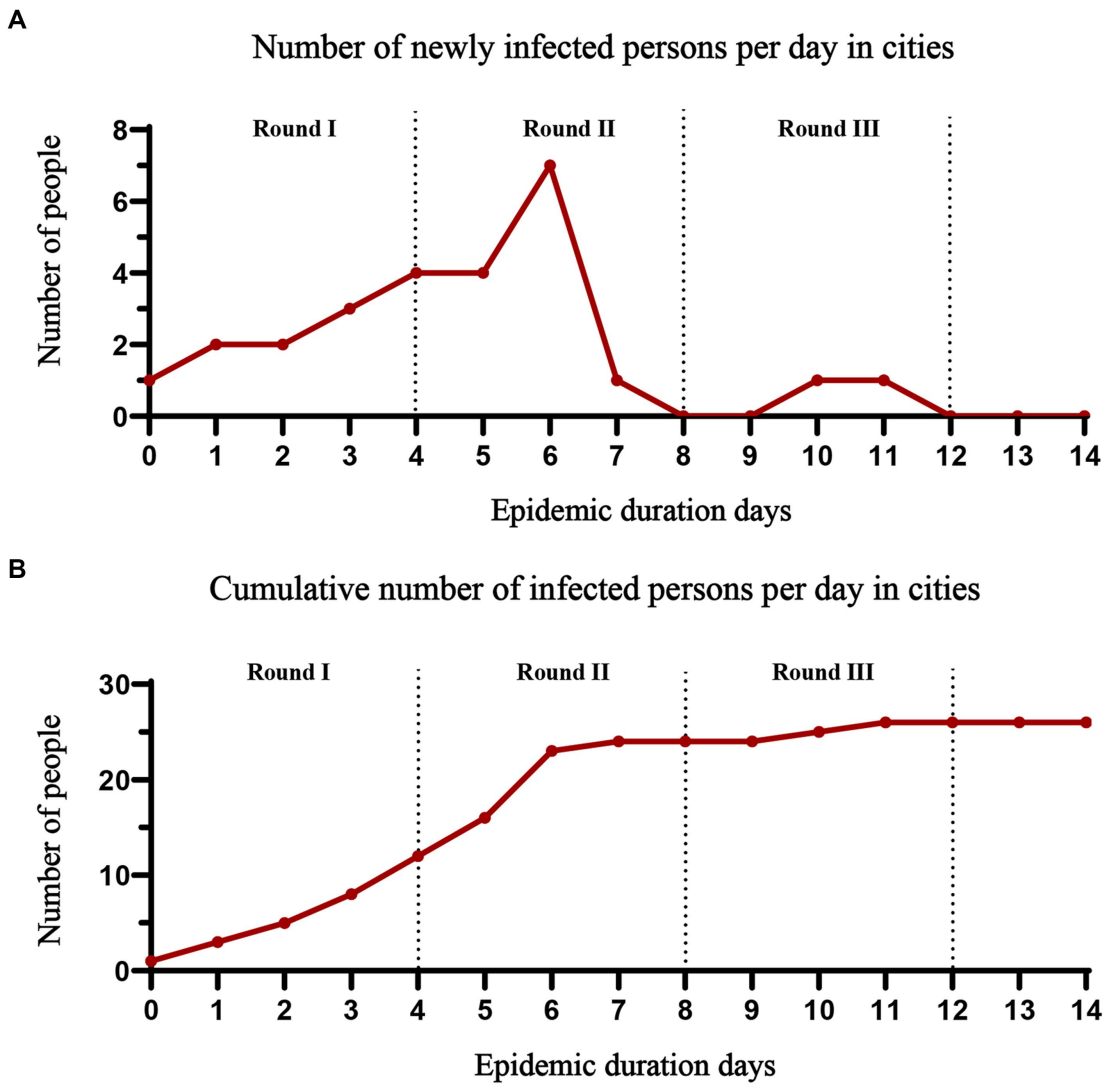
The trajectory of the number of people infected with the epidemic. **(A)** Daily changes in the number of new infections; **(B)** Total number of infected individuals.

Most of the infected persons were found in the first two space–time companion rounds (8 days). According to our data, the spread of the epidemic has been controlled after three rounds. The curves of daily new infections and total infections per day during the small outbreak of the epidemic are shown in Figure 3.

## Discussion

In this preliminary study, we investigated the process and results of the space–time companion epidemic management method during an epidemic outbreak in Chengdu, China. Given the findings reported in our study, we aimed to provide a new method for screening close contacts of COVID-19 infectors and other similar infectious diseases.

The ideal strategy for epidemic prevention and control must not only stop the spread; however, it should also minimize the number and scale of affected citizens. The management policy based on Health QR Code system has proven to be accurate and feasible to a certain extent in the case of sporadic or small-scale epidemic situations (9, 25, 28). However, a more accurate determination of the yellow and red codes is necessary. The previous determination method was an oversaturation strategy. For example, on July 17, 2021, after three newly confirmed cases were found in Zhangjiajie, the government blocked and isolated all people in Zhangjiajie, including approximately 11,900 tourists and tested for nucleic acid, which may be an excessive epidemic prevention strategy to a certain extent (29). Owing to the excessive number of stranded people, allocation of living resources, insufficient capacity of the local medical and epidemic prevention system, and possible shortage of human, material, and medical resources, resulted in the consumption of unnecessary human and social resources. With the emergence of the concept of space–time companion, the government can screen relevant personnel for space–time companions and accurately identify possible infected persons. The orderly evacuation of other tourists can relieve the work pressure of epidemic prevention and control officers, and improve the efficiency of epidemic prevention.

Traditional epidemiological investigation methods may have the risk of consuming human and material resources, further spreading the epidemic over a long period of time, and spreading the epidemic through personnel concealment and omission (30–32). In addition, studies have shown that most people infected with the delta variant do not have obvious symptoms such as fever, and even asymptomatic transmission could occur in the population who have been vaccinated with the new crown vaccine (33, 34). If someone deliberately misrepresents or conceals the itinerary, there may be a very serious epidemic transmission chain. Therefore, screening infected persons are an important problem in the epidemic prevention and control of COVID-19. After the outbreak of the epidemic in Nanjing, a 64-year-old woman left the control area without permission. She left Nanjing to Yangzhou City, concealing the history of high-risk areas and her trip, which led to the spread of COVID-19 in Yangzhou (7, 35, 36). Compared with traditional epidemiological investigation methods, space–time companions can quickly and accurately screen risk personnel, and can quickly and effectively control the spread of the epidemic. Meanwhile, some artificial intelligence-based algorithm models can also perform pattern recognition of infection for spatiotemporal distribution assessment and spread pattern© analysis, to predict the spread trend of the epidemic (37, 38).

The rapid control of the epidemic in Chengdu, Sichuan, benefited from previous experiences in epidemic prevention, such as the arrangement of epidemic prevention experts for epidemic prevention and control and the public’s cooperation in epidemic prevention and control measures. In contrast to the past, space–time companion management procedures can screen out risk personnel faster and more accurately, implement isolation measures on a smaller scale, reduce the pressure of epidemic prevention and control, and increase efficiency.

This preliminary study adds to our understanding of the epidemiological history survey under COVID-19; however, it has numerous limitations that should be considered. First, the effectiveness of this mode needs to be confirmed using more future data. The epidemic prevention and control situation in Chengdu, China alone is not sufficient to prove its effectiveness. Second, this method is only applicable to places with good network coverage. Although 4G signal coverage has been achieved in most parts of China, there are still special environments with poor signals, such as underground buildings, tunnels, and other areas. Ensuring the effectiveness of the space–time companion mode in such places may require more accurate algorithms and other methods. Third, this method temporarily needs to be used in conjunction with the three-color Health Code System to narrow the screening range and avoid intentional escape from surveillance. The three-color Health Code System require people use their mobile phones to show their green health code when entering any public place (such as a company or community), to prevent anyone from using shutdown methods to escape monitoring. Meanwhile, through this system, the screening scope can be reduced. If the activity scope of a certain space–time companion is only within the community, the screening scope of 800 * 800 m will be limited to the community. And if a space–time companion has not entered the community, those who have not gone out from the community during this period will not be considered space–time companions, even if they are within the range of 800 * 800 m. Notably, the life of yellow and red code people under the three-color Health Code System may be affected. Under the compulsory isolation measures for these people under the epidemic prevention and control in China, their work and life may be inconvenient, especially in places with improper allocation of social service personnel, too many space–time companions may consume too much human and social resources. Therefore, this method is only applicable in the early stages of the epidemic or when the epidemic is preliminarily controlled.

Based on the above reasons, this method cannot be used as a separate screening method for close contacts temporarily, but can be used as a supplement and verification to existing epidemiological history investigations. Moreover, it can only be used in areas with full network coverage and conjunction with three-color Health Code System to narrow the screening scope and improve accuracy. However, exploring a new epidemiological investigation method to find and exclude close contacts in a more timesaving, labor-saving, and comprehensive way is necessary to prepare for a possible epidemic of mutated or other virus in the future. Besides, with the further development of communication technology, this method will inevitably be more widely applied and play a greater role in epidemiological history investigations.

## Conclusion

In conclusion, the space–time companion provides a new idea for screening close contacts of the COVID-19 infector and other similar infectious diseases, which may be the turning point from traditional artificial epidemiology investigation to intelligent models.

## Data availability statement

The original contributions presented in the study are included in the article/supplementary material, further inquiries can be directed to the corresponding authors.

## Ethics statement

The study has been approved by the Ethics Committee of the Chengdu Women’s and Children’s Central Hospital (No. 202039).

## Author contributions

All authors listed have made a substantial, direct, and intellectual contribution to the work and approved it for publication.

## Funding

Financial support for this work was provided by the Sichuan Provincial Department of Science and Technology (2023YFS0219 and 2023YFS0228), and Chengdu Science and Technology Bureau (22ZDYF1597 and 2021-YF09-00048-SN). The funding agencies did not have any role in the design of the study, collection, analysis, and interpretation of data, or in writing the manuscript.

## Conflict of interest

The authors declare that the research was conducted in the absence of any commercial or financial relationships that could be construed as a potential conflict of interest.

## Publisher’s note

All claims expressed in this article are solely those of the authors and do not necessarily represent those of their affiliated organizations, or those of the publisher, the editors and the reviewers. Any product that may be evaluated in this article, or claim that may be made by its manufacturer, is not guaranteed or endorsed by the publisher.

## Footnotes

^1^ http://www.nhc.gov.cn/xcs/yqtb/list_gzbd.shtml
